# Comparing rates of clinically relevant epistaxis in patients taking warfarin versus direct oral anticoagulants

**DOI:** 10.1016/j.rpth.2024.102630

**Published:** 2024-11-20

**Authors:** Khristian S. Burke, Xiaowen Kong, Brian Haymart, Debbie DeCamillo, Mona Ali, Geoff Barnes, Scott Kaatz

**Affiliations:** 1 Central Michigan University College of Medicine, Jeddo, Michigan, USA; 2 Department of Internal Medicine, University of Michigan, Frankel Cardiovascular Center, Ann Arbor, Michigan, USA; 3 Department of Heart and Vascular Services, Corewell Health William Beaumont University Hospital, Royal Oak, Michigan, USA; 4 Division of Hospital Medicine, Department of Internal Medicine, Henry Ford Health, Detroit, Michigan, USA

## 1 Introduction

Epistaxis requiring emergency department (ED) treatment is a common bleeding complication in patients taking anticoagulants, accounting for 8% to 16% of treatment-emergent adverse events [1,2]. Limited existing evidence comparing epistaxis events between warfarin and direct oral anticoagulants (DOACs) has been mixed. The Rivaroxaban Once Daily Oral Direct Factor Xa Inhibition compared with Vitamin K Antagonism for Prevention of Stroke and Embolism Trial (ROCKET AF) showed rivaroxaban to have more clinically relevant epistaxis events compared with warfarin, with similar findings in the Japanese ROCKET AF (J-ROCKET AF) trial, although the results were not assessed for statistical significance [1,2]. Conversely, a recent nationwide Icelandic cohort study reported higher rates of epistaxis in patients taking warfarin when compared with DOACs [3].

The purpose of this study was to compare rates of ED visits for epistaxis between patients taking warfarin vs DOACs for atrial fibrillation (AF) or venous thromboembolism (VTE) enrolled in the Michigan Anticoagulation Quality Improvement Initiative (MAQI^2^) registry.

## 2 Methods

### 2.1 Data source

MAQI^2^ is a Blue Cross Blue Shield of Michigan-funded quality improvement registry of patients taking oral anticoagulation medications from 6 health systems across Michigan, which began patient enrollment in 2009 [4].

Data are retrospectively collected and analyzed from cohorts of patients on warfarin or DOAC therapy, starting from the time of enrollment through the earliest of the last follow-up before May 31, 2022, treatment discontinuation, or death. Data collection is performed by trained abstractors and verified by periodic audits, ensuring agreement with predefined data element definitions.

### 2.2 Patient population

Patients on warfarin were included from October 2009 to May 2022. Patients on DOACs were included from November 2015 to May 2022. Exclusion criteria included long-term warfarin therapy prior to MAQI^2^ enrollment or a primary indication for anticoagulation other than AF or VTE. Because patients enrolled in the MAQI^2^ DOAC registry could not be on any oral anticoagulant for at least 1 year prior to enrollment, no exclusion criteria for long-term oral anticoagulant use were necessary for this group.

### 2.3 Data collection and outcomes

Baseline data were collected at the time of enrollment. Outcome data were abstracted at each anticoagulation services interaction for warfarin patients or every 6 months via chart reviews for those on DOACs.

The primary outcome was the rate of clinically relevant epistaxis events, defined as epistaxis requiring ED evaluation and treatment. This incorporated major and nonmajor bleeding events. Secondary outcomes included median days from enrollment to the first epistaxis event, epistaxis events meeting criteria for major bleeding as defined by the International Society on Thrombosis and Haemostasis [5], hospital admissions for epistaxis, and recurrent epistaxis. The MAQI^2^ DOAC registry did not record the number of hospital days per admission and was not compared between groups.

### 2.4 Statistical analysis

Baseline information was compared using Student’s *t*-tests for continuous variables and chi-squared and Fisher’s exact tests for categorical variables. Inverse probability weighting (IPW) was used to mitigate differences between groups. Variables included in the IPW model included age, biological sex assigned at birth, non-Hispanic White, modified hypertension, abnormal renal/liver function, stroke, bleeding history of disposition, labile international normalized ratio, elderly (>65 years), drugs/alcohol concomitantly (HAS-BLED) score, comorbid conditions, concomitant antiplatelet or nonsteroidal anti-inflammatory drug (NSAID) use, and treatment indication. A modified HAS-BLED score was calculated for each patient at the time of enrollment, removing the labile international normalized ratio (INR) variable.

Poisson regression was utilized to compare outcomes after IPW. A 2-sided *P* <.05 was considered statistically significant for all analyses. Analyses were performed using SAS version 9.4 and R-64 4.1.3 software, developed by SAS Institute and the R Development Core Team, respectively.

## 3 Results

### 3.1 Participant data

After exclusions, a total of 10,557 and 3889 patients met inclusion for the warfarin and DOAC groups, respectively. In the DOAC group, 2750 (70.9%) patients received apixaban, 1105 (28.5%) received rivaroxaban, 23 (0.59%) received dabigatran, 3 (0.08%) received edoxaban, and 7 had no data on which DOAC was used. Several baseline characteristics were significantly different between cohorts. However, the IPW model demonstrated all standardized differences to be <0.1 (Table 1).

**Table 1:** Patient characteristics.

	Warfarin (*N* = 10,557)	DOACs (*N* = 3889)	*P* value
**Demographics**	-	-	-
Male sex	5564 (52.7)	1913 (49.2)	.0002
Age (y), mean (SD)	65.3 (15.4)	68 (14.4)	<.001
Non-Hispanic White	8058 (76.3)	3069 (78.9)	.001
Black	1702 (16.1)	608 (15.6)	<.001
Asian	104 (1)	55 (1.4)	<.001
Native American	29 (0.3)	7 (0.2)	<.001
Hispanic	78 (0.7)	16 (0.4)	<.001
Other	586 (5.6)	134 (3.5)	<.001
**Comorbidities**	-	-	-
Hypertension	6941 (65.8)	2784 (71.6)	<.0001
Diabetes mellitus	2717 (25.7)	1029 (26.5)	.38
Congestive heart failure	1992 (18.9)	624 (16.1)	<.0001
Peripheral vascular disease	576 (5.5)	188 (4.8)	.14
Alcohol use	539 (5.1)	236 (6.1)	.02
Recent bleeding history	291 (2.8)	141 (3.6)	.007
Remote bleeding history	347 (3.3)	224 (5.8)	<.0001
Chronic liver failure	267 (2.5)	170 (4.4)	<.0001
Bleeding diathesis	53 (0.5)	4 (0.1)	.001
**Concomitant medications**	-	-	-
NSAIDs	408 (3.9)	128 (3.3)	.11
Antiplatelets	4430 (42)	1323 (34)	<.0001
**Bleeding risk**	-	-	-
Modified HAS-BLED score, mean (SD)	2.5 (1.4)	2.5 (1.4)	.12
**Treatment indication**	-	-	-
AF	5735 (54.3)	2337 (60.1)	<.0001
VTE	4948 (46.9)	1587 (40.8)	<.0001
**Anticoagulant**	-	-	-
Dabigatran	N/A	23 (0.59)	N/A
Rivaroxaban	N/A	1105 (28.5)	N/A
Apixaban	N/A	2750 (70.9)	N/A
Edoxaban	N/A	3 (0.08)	N/A
Warfarin	10,557	N/A	N/A

Data are presented as *n* (%) unless otherwise specified.

AF, atrial fibrillation; DOAC, direct oral anticoagulant; HAS-BLED, hypertension, abnormal renal/liver function, stroke, bleeding history of disposition, labile international normalized ratio, elderly (>65 years), drugs/alcohol concomitantly; N/a, not applicable; NSAID, nonsteroidal anti-inflammatory drug; VTE, venous thromboembolism.

### 3.2 Outcome data

Patients receiving warfarin had significantly higher rates of clinically relevant epistaxis than those on DOACs, with an observed rate of 2.06 events per 100 patient-years (events/100-py) vs 0.75 events/100-py and an estimated rate of 2.02 events/100-py (95% CI, 1.8, 2.2) vs 0.73 events/100-py (95% CI, 0.6, 1.0); *P* < .0001.

Of 378 ED visits in the warfarin group, 18 (4.8%) met the criteria for major bleeding, and 93 (24.6%) resulted in hospital admission. Of 57 ED visits in the DOAC group, 3 (5.3%) met the criteria for major bleeding, and 10 (17.5%) resulted in hospital admission. Patients on warfarin had higher observed and estimated rates of hospital admission for epistaxis than patients on DOACs (observed rate 0.50 events/100-py vs 0.13 events/100-py; estimated rate 0.49 events/100-py [95% CI, 0.41, 0.58] vs 0.14 events/100-py [95% CI, 0.098, 0.019]; *P* < .0001), as well as higher observed and estimated rates of epistaxis meeting major bleed criteria (observed rate 0.098 events/100-py vs 0.04 events/100-py; estimated rate 0.096 events/100-py [95% CI, 0.065, 0.143] vs 0.034 events/100-py [95% CI, 0.018, 0.065]; *P* < .007). Primary and secondary outcome data are shown in Table 2.

**Table 2:** Outcomes.

	Warfarin *N* = 10,557	DOACs *N* = 3889	*P* value
Median years of follow-up (IQR)	0.59 (2.1)	1.5 (2.5)	-
Median days from enrollment to first epistaxis event (IQR)	483 (864)	369 (719)	.28
ED visits for epistaxis, *n*	378	57	-
Major epistaxis events	18 (4.8)	3 (5.3)	-
Hospital admissions	93 (24.6)	10 (17.5)	-
Outcomes in the no. of events per 100 patient-years of follow-up (95% CI)			
ED visits for epistaxis – observed	2.06	0.75	-
ED visits for epistaxis – estimated	2.02 (1.86-2.21)	0.73 (0.63-0.083)	<.0001
Major epistaxis – observed	0.098	0.04	-
Major epistaxis – estimated	0.096 (0.065-0.143)	0.034 (0.018-0.065)	<.007
Hospital admissions – observed	0.50	0.13	-
Hospital admissions – estimated	0.49 (0.41-0.58)	0.14 (0.098-0.019)	<.0001

Data are presented as *n* (%) unless otherwise specified.

DOAC, direct oral anticoagulant; ED, emergency department.

## 4 Discussion

Our observational cohort study showed that patients receiving warfarin for AF or VTE had significantly higher rates of clinically relevant epistaxis compared with patients taking DOACs, aligning with a recent study in Icelandic patients [3] but contrasting with others [1,2].

It is unclear why event rates differ between studies. One reason could be differences in epistaxis rates between DOACs. More than 70% of our study population was taking apixaban, which is associated with lower bleeding rates than other DOACs when compared with warfarin [2,6]. The limited existing data have also shown that rivaroxaban has higher epistaxis rates than apixaban [3]. Another reason could be the intensity of anticoagulation at the time of the event. Since INR values could not be compared between groups, they were not included in this study. It is unclear if patients on warfarin had supratherapeutic INRs at the time of ED presentation. Studies including INR values in patients taking vitamin K antagonists have shown average INR values to be mostly therapeutic upon ED presentation [3,7,8], while another found most patients to be supratherapeutic [9]. The differences in epistaxis event rates may be multifactorial: a combination of differences in event rates between DOACs and the intensity of anticoagulation at the time of the epistaxis event.

This study also shows that patients on warfarin have higher rates of epistaxis meeting criteria for major bleeding and hospital admissions compared with DOACs. This adds to previous literature, which has shown mixed results [3,8]. Again, this difference is likely multifactorial and may be impacted by our DOAC group being primarily on apixaban.

There are notable limitations to our study. Our study population was restricted to the state of Michigan within anticoagulation clinics participating in the MAQI^2^ initiative. However, our study population was 21% to 24% non-White, whereas similar studies had primarily homogenous, non-Hispanic, White study populations [3,8].

Given the retrospective and observational nature of this registry, there is a risk of confounding bias as patients were not randomized. Reasonable measures were taken to correct for confounding, illustrated by the IPW model demonstrating all weighted standardized differences to be <0.1.

This study highlights the importance of efforts to reduce epistaxis and health care utilization in patients on oral anticoagulants. In patients with an increased risk of epistaxis, switching from warfarin to a DOAC may be beneficial if no contraindications exist. Efforts should aim to reduce preventable ED visits for epistaxis in DOAC patients, such as through educational programs similar to those for warfarin [10]. Additional research to further characterize differences in epistaxis rates between DOACs and identify strategies to reduce epistaxis-related ED visits is needed.
